# Cancer-associated fibroblasts rewire the estrogen receptor response in luminal breast cancer, enabling estrogen independence

**DOI:** 10.1038/s41388-024-02973-x

**Published:** 2024-02-22

**Authors:** Steven E. Reid, Jessica Pantaleo, Paulina Bolivar, Matteo Bocci, Jonas Sjölund, Mikkel Morsing, Eugenia Cordero, Sara Larsson, Maria Malmberg, Brinton Seashore-Ludlow, Kristian Pietras

**Affiliations:** 1 Department of Laboratory Medicine, Division of Translational Cancer Research, Lund University Cancer Centre, Medicon Village, Lund University, Lund, Sweden; 2 Department of Oncology-Pathology, SciLifeLab, Stockholm, Sweden; 3 Chemical Biology Consortium Sweden (CBCS), Karolinska Institute, Stockholm, Sweden

**Keywords:** Breast cancer, Cancer microenvironment, Mechanisms of disease

## Abstract

Advanced breast cancers represent a major therapeutic challenge due to their refractoriness to treatment. Cancer-associated fibroblasts (CAFs) are the most abundant constituents of the tumor microenvironment and have been linked to most hallmarks of cancer. However, the influence of CAFs on therapeutic outcome remains largely unchartered. Here, we reveal that spatial coincidence of abundant CAF infiltration with malignant cells was associated with reduced estrogen receptor (ER)-α expression and activity in luminal breast tumors. Notably, CAFs mediated estrogen-independent tumor growth by selectively regulating ER-α signaling. Whereas most prototypical estrogen-responsive genes were suppressed, CAFs maintained gene expression related to therapeutic resistance, basal-like differentiation, and invasion. A functional drug screen in co-cultures identified effector pathways involved in the CAF-induced regulation of ER-α signaling. Among these, the Transforming Growth Factor-β and the Janus kinase signaling cascades were validated as actionable targets to counteract the CAF-induced modulation of ER-α activity. Finally, genes that were downregulated in cancer cells by CAFs were predictive of poor response to endocrine treatment. In conclusion, our work reveals that CAFs directly control the luminal breast cancer phenotype by selectively modulating ER-α expression and transcriptional function, and further proposes novel targets to disrupt the crosstalk between CAFs and tumor cells to reinstate treatment response to endocrine therapy in patients.

## Introduction

It is now widely accepted that a tumor is built through paracrine interactions between malignant cells and their microenvironment. Reciprocal signaling involving cancer cells, immune cells, vascular cells, and cancer-associated fibroblasts (CAFs) promote tumor initiation, expansion, and dissemination. In addition, the tumor microenvironment (TME) is instrumental in shaping the response to both conventional and targeted therapies. Specifically, we and others have previously demonstrated that CAFs dampen the effect of chemotherapy by engineering a high interstitial fluid pressure and a physical collagen barrier that limit the influx of drug from the vasculature to the tumor parenchyma [1–4]. Similarly, CAFs may reduce the efficacy of both external beam radiotherapy and radioimmunotherapy [5, 6]. More recently, CAFs have been described as immunosuppressive, and depletion of CAF subsets expressing fibroblast activation protein (FAP)-α or α-smooth muscle actin (SMA) sensitizes experimental tumors to immune checkpoint blockade with α-CTLA-4 or α-PD-L1 [7, 8]. Taken together, CAFs emerge as important regulators of drug response to mainstay anti-cancer treatment modalities in a range of malignant diseases, and development of strategies to counteract their contribution to therapeutic resistance are warranted to improve treatment benefit.

Treatment of breast cancer is dictated by the molecular subtype of the disease. Whereas luminal, hormone receptor-expressing tumors, are effectively managed by endocrine therapies at early stages, and patients with HER2-amplified tumors benefit from treatment with HER2-targeted therapies, treatment options for triple-negative breast cancers (TNBC) remain limited to chemo- and radiotherapy [9]. Recently, we demonstrated that the TNBC phenotype is in part microenvironmentally maintained by the paracrine action of platelet-derived growth factor (PDGF)-CC expressed by malignant cells, signaling to CAFs that reciprocate with a cocktail of hepatocyte growth factor (HGF), insulin-like growth factor binding protein (IGFBP) 3, and stanniocalcin (STC)1. Combined, these factors dictate a global down-regulation of the luminal gene expression program, including ER-α and its pioneering transcription factor FoxA1 [10]. Notably, genetic or pharmacological ablation of PDGF-CC activity converted experimental TNBC to an ER-α^+^ subtype that was amenable to endocrine therapy with tamoxifen. Conversely, ectopic expression of PDGF-CC in luminal breast cancer cells conferred resistance to tamoxifen treatment. However, the mechanistic underpinnings and the generality of the regulation of the molecular subtype of breast cancer by CAFs remain largely unexplored.

Here, we have utilized co-cultures and orthotopic co-transplantation models of luminal breast cancer with CAFs to delineate conceptual and molecular mechanisms of the paracrine regulation of ER-α signaling. Histological analysis demonstrated a reduced expression of ER-α in areas of experimental breast tumors with pronounced stromal invasion. Intriguingly, we found that CAFs modulate the ER-α-induced transcriptome in a selective manner, conferring estrogen-independent growth in vivo by dampening much of the classical hormone-induced gene expression program, whilst retaining several hallmark pro-tumoral signaling pathways involved in basal-like differentiation, tumor invasiveness and therapeutic resistance. A functional drug screen using co-culture of breast cancer cells and CAFs revealed molecular signaling pathways contributing to the regulation of ER-α-signaling by CAFs, suggesting TGF-β and JAK signaling as potential nodes for intervention to counteract the paracrine regulation. Taken together, we shine further light on how CAFs modify the transcription of genes involved in endocrine therapy resistance of breast cancer cells; CAFs emerge as important regulators of endocrine therapy resistance and the TNBC phenotype, and CAF targeting strategies should be considered as adjuvants to hormonal therapy.

## Results

### Estrogen receptor expression in tumor cells is inversely correlated with stromal content

**Fig. 1:**
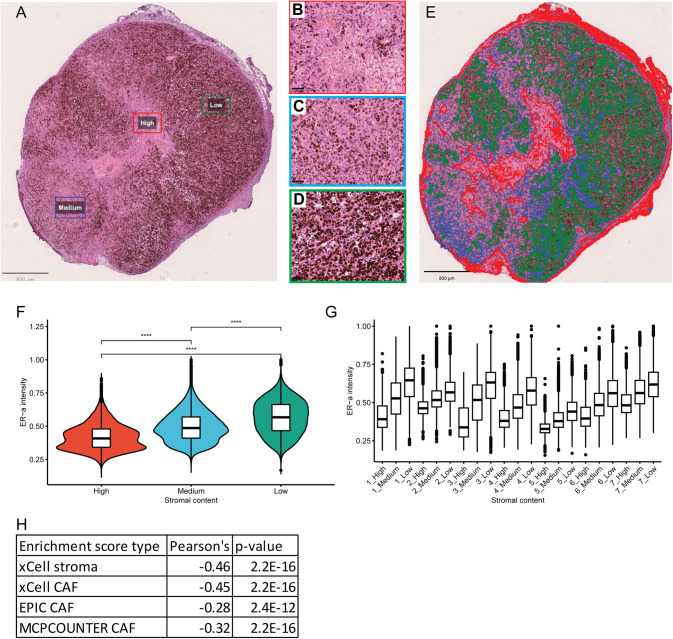
Breast cancer cells exhibit reduced ER-α expression in high stromal regions. Tumors were stained for ER-α (**A**) and compartmentalized into defined areas of high (**B**; red border), medium (**C**; blue border) or low (**D**; green border) stromal infiltration, as demonstrated for a full tumor in (**E**). The DAB staining intensity was measured for each cell; the mean for all measured areas is shown in (**F**), and for each individual tumor compartmentalized according to stromal intensity in (**G**). Unpaired two-tailed t-test ****P < 0.0001. Error bars: SEM, data from *n* = 7 tumors. Cell enrichment scores for stroma or CAFs from different bioinformatic tools enable correlation of CAF content with *ESR1* expression (**H**).

We have previously demonstrated that CAF-derived factors can decrease the expression of ER-α in breast tumors to induce a triple-negative phenotype [10]. Here, we sought to identify whether ER-α expression correlated to the local abundance of stroma within breast tumors. To this end, we analyzed the intensity of expression of human ER-α, as visualized by immunohistochemical staining of sections from orthotopically xenografted MCF7 tumors, in coherent spatial environments histologically classified as having a low ( ≥ 70% ER-α^+^ cells), medium (26–69% ER-α^+^ cells) or high ( ≤ 25% ER-α^+^ cells) stromal infiltration pattern by a trained cell classifier (Fig. 1A–E). Intriguingly, using a cell intensity-based measurement, where the average intensity of the ER-α staining within each cell was plotted in the three regions, stromal content was found to inversely correlate to ER-α-positivity such that the lowest expression was found in areas of high stromal infiltration, suggestive of paracrine regulation (Fig. 1F, G).

To corroborate our findings in specimens from human breast cancer patients, we analyzed RNA-seq data from all tumors classified as Luminal A or B in the TCGA BRCA cohort. By using several different cell deconvolution algorithms, including xCEll, MCP-counter and EPIC, to derive cell type enrichment scores, we found that the expression of *ESR1* consistently exhibited an inverse correlation with the abundance of CAFs (Fig. 1H), consistent with our findings in experimental tumors. Taken together, we conclude that the abundance of CAFs correlates with a lower expression of ER-α in luminal breast cancers.

### Cancer-associated fibroblasts reduce estrogen receptor expression and activity in breast cancer cells

**Fig. 2:**
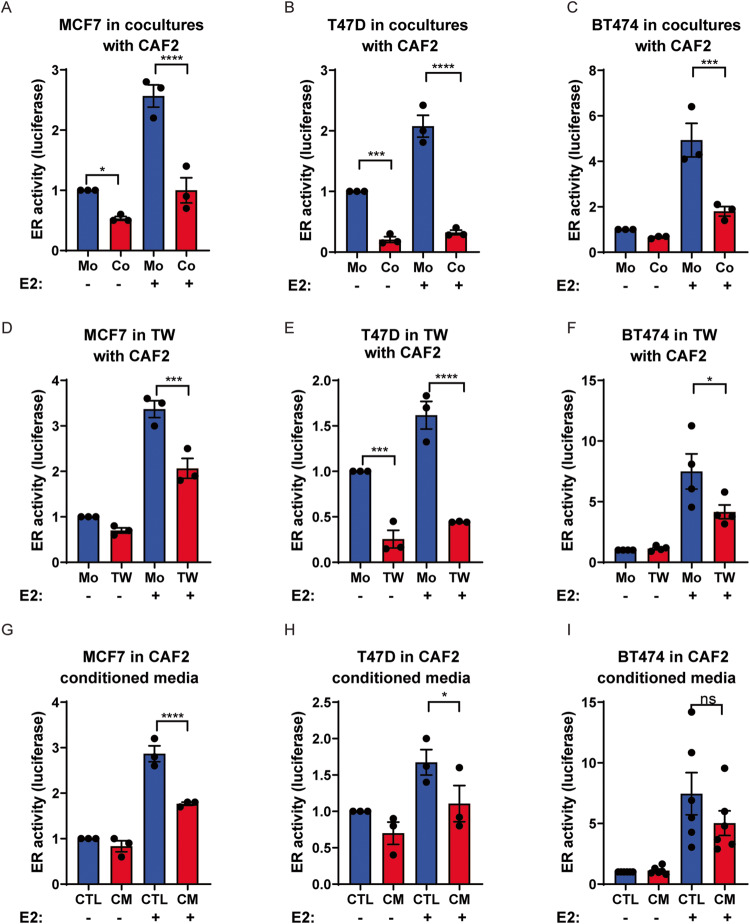
CAFs reduce estrogen receptor activity in co-cultures in part via secreted factors. The CAF2 cell line affects ER-α activity in luminal breast cancer cell lines MCF7, T47D and BT474 clone 5 cells in direct co-cultures for 96 h (**A**–**C**), in transwell (TW) co-cultures for 72 h (**D**–**F**) and in CAF2-conditioned media for 48 h (**G**–**I**). MCF7 cells were starved of estrogen for 24 h before stimulation with estrogen for 48 h and ER-α-induced luciferase activity measured. Mo = monoculture; Co = co-culture; CM: CAF2 Conditioned Media, E2 = estrogen. Unpaired ordinary one-away ANOVA, Fisher’s LSD multiple comparisons test: *P < 0.05, ***P < 0.001, ****P < 0.0001, ns not significant. Error bars: SEM.

**Fig. 3:**
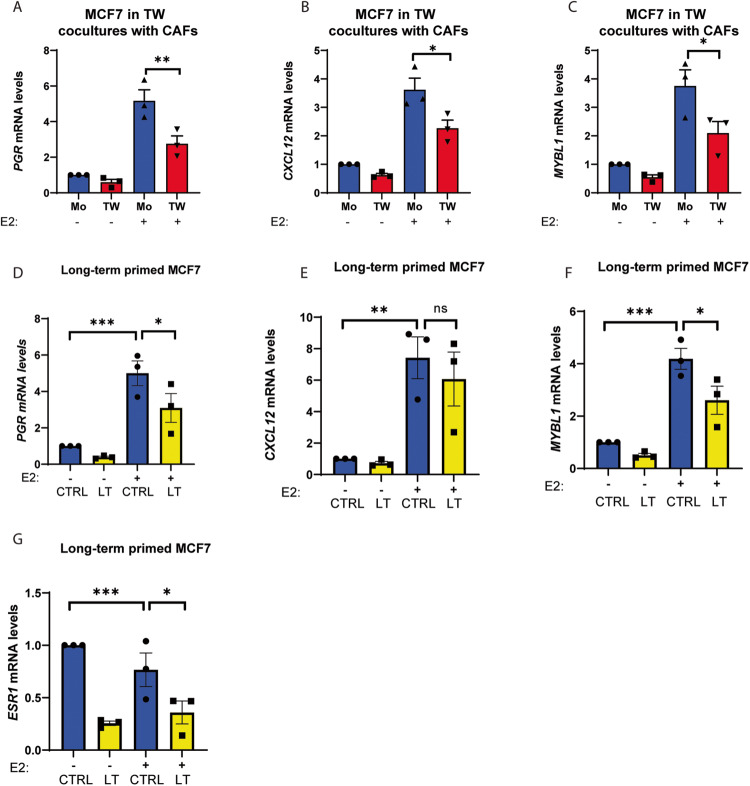
CAFs reduce ER target gene expression in MCF7 cells via secreted factors. Induction of ER-α target genes *PGR*, *CXCL12* and *MYBL1* by estrogen (E2) in MCF7 cells in mono-culture or co-cultured with CAF2 for 48 h in transwells (TW) (**A**–**C**). Induction of the same genes of control (Ctrl) MCF7 cells or those that had previously been primed by CAF2 in transwells long-term (**D**–**F**). Transcription of *ESR1* mRNA is reduced in long-term conditioned MCF7 cells (**G**). RNA levels are normalized to the untreated monocultures (Mo) or Ctrl MCF7 levels. E2 = estrogen. Unpaired ordinary one-way ANOVA, Fisher’s LSD multiple comparisons test: *P < 0.05, **P < 0.01, ***P < 0.001. Error bars: SEM.

Since local stromal content correlated inversely with ER-α expression in MCF7 tumors, we sought to identify the impact of CAFs on ER-α transcriptional activity in human breast cancer cells in a co-culture model. To this end, we engineered three ER-α-expressing human breast cancer cell lines with a luciferase reporter construct under the control of three consecutive Estrogen Response Elements (ERE) to generate ER-α reporter cell lines: MCF7 (ER-α^+^; PR^+^), T47D (ER-α^+^; PR^+^), and BT474 clone 5 (ER-α^+^; PR^-^ ; HER2^+^). Strikingly, breast cancer cells in direct co-culture with an immortalized human breast CAF cell line engineered through in vivo conditioning by MCF7 cells (CAF2, CAF:BC ratio 3:1) [11] exhibited a significantly muted ER-α activity following stimulation with estrogen (E2) in all three breast cancer cell lines, compared to mono-cultures of malignant cells (Fig. 2A–C). Moreover, CAF2 reduced the basal ER-α activity even without supplemented estrogen in MCF7 and T47D cells (Fig. 2A, B). The suppressive effect of CAFs on MCF7 ERE activity was confirmed using two separate immortalized CAF lines isolated from a human breast tumor (SFig. 1A). The reduced ERE activity was accompanied by a downregulation of ER-α protein expression in MCF7 cells, as assessed by immunoblotting (SFig. 1B, C). To understand whether physical cell contact was required for the heterotypic interaction, we also assessed ERE activity in breast cancer cells that were cultured together with CAF2 physically separated in transwells. The ER-α activity of all three breast cancer lines was reduced in transwell cultures with the CAF2 cell line (Fig. 2D–F). Finally, to evaluate whether unidirectional signaling from the CAF2 to the breast cancer cells was sufficient to suppress estrogen signaling, we measured ER-α activity in breast cancer cell lines cultured in concentrated CAF2-conditioned media. Unidirectional CAF2 signaling through soluble factors was indeed able to decrease ERE activity in both MCF7 and T47D cell lines, yet in BT474 this did not reach statistical significance (Fig. 2G–I). To verify that the ERE transcriptional activity was reflective of actual target gene expression, we further assessed the expression of the ER-α target genes *PGR*, *CXCL12* and *MYBL1* [12–15] following estrogen stimulation in the MCF7 cell line in transwells with CAF2. The three ER-α target genes were expressed at significantly lower levels in CAF2 transwell co-cultures compared to estrogen stimulation in the monocultures (Fig. 3A–C). Interestingly, suppression of HER2 expression induced by estrogen in the HER2^+^ BT474 clone 5 cells was not affected by the paracrine action of CAF2, indicating that regulation of ER-α target genes by CAF2 may be selective (SFig. 2A, B). In addition, we used MCF7 cells that had been serially cultured either with or without CAF2 in transwells for 50 passages over the course of one year to generate new sublines termed long-term (LT) primed MCF7 cells, then assessed ER-α expression and target gene expression (Fig. 3D–G). Here, two of the three target genes were also reduced in the LT CAF2-primed MCF7, even after CAF2 were no longer present (Fig. 3D–F). In addition, *ESR1* itself was reduced in the LT cells and the *ESR1* transcription level was no longer responsive to estrogen (Fig. 3G). These data suggest that CAF2 have a lasting effect on the MCF7 cultures, either through epigenetic regulation or through selection of ER-α-low subclones over time.

### Cancer-associated fibroblasts confer growth to orthotopic luminal breast cancer xenografts under estrogen-limiting conditions

**Fig. 4:**
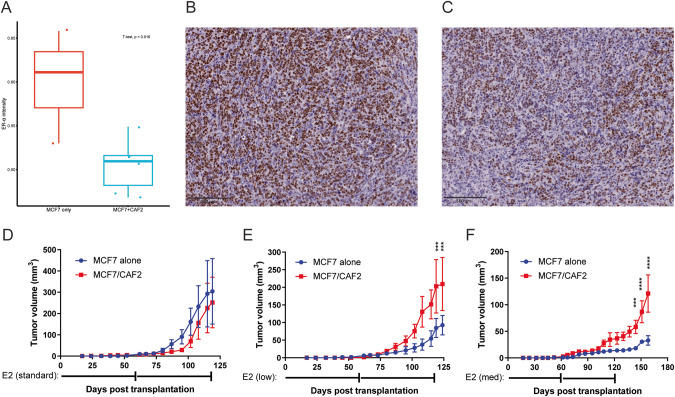
CAF co-implantation enables breast tumor formation in limited estrogen availability. Quantification of the immunohistochemistry staining intensity for ER-α (**A**) of tissue sections from MCF7 tumors established without (**B**) or with (**C**) the support of CAF2 cells. Unpaired, two-sided Student’s t-test *P < 0.05. Tumor growth curves in mice either transplanted with MCF7 cells alone or in combination with CAF2 cells in the mammary fat pad with standard (0.5 mg) (**D**) or low (0.025 mg) (**E**) dose estrogen levels. The 60-day slow-release pellets were replenished as indicated below the x-axis. Tumor growth in the presence of an intermediate estrogen dose (0.1 mg) (**F**) was continually monitored without further estrogen supplementation. E2 = estrogen. Multiple unpaired, two-sided t-tests (Two-stage step-up (Benjamini, Krieger, and Yekutieli): ***P < 0.001, ****P < 0.0001. Error bars: SEM; *n* = 5 mice in each of the six groups.

Based on our observations that CAF2 modulates ER-α expression in vivo and estrogen receptor expression and activity in luminal breast cancer cells in vitro, we sought to determine whether CAF2 regulates estrogen-dependence in vivo during tumor initiation and growth. To this end, we transplanted MCF7 cells orthotopically with, or without, supportive CAF2 cells. Tumors that developed through co-transplantation of MCF7 and CAF2 cells exhibited an overall lower intensity of ER-α immunostaining, compared to tumors resulting from transplantation of MCF7 cells alone (Fig. 4A–C). In the presence of the standard concentration of estrogen supplied by a slow-release pellet (0.5 mg released over 60 days), MCF7 cells readily formed tumors with the same latency and growth rate, regardless of CAF2 support (Fig. 4D). In contrast, at a low and limiting concentration of estrogen (0.025 mg released over 60 days), CAF2 conferred a significant growth advantage to co-transplanted MCF7 cells, indicating that paracrine support from CAF2 relieve estrogen-dependency of luminal breast cancer cells (Fig. 4E). Supplying an intermediate dose of estrogen (0.1 mg released over 60 days) for 120 days to initiate tumor growth, and subsequently depriving hormonal support altogether, further demonstrated the ability of CAF2 to grant estrogen-independent growth to MCF7 tumors, in contrast to the unsupported breast tumors that did not grow appreciably following discontinuation of estrogen to established tumors (Fig. 4F). Taken together, the in vivo co-transplantation experiments illustrate that CAFs reduce the estrogen dependency of luminal breast cancer cells through paracrine support, the molecular nature of which we sought to determine through gene expression analyses.

### Cancer-associated fibroblasts modulate estrogen signaling in a selective manner

**Fig. 5:**
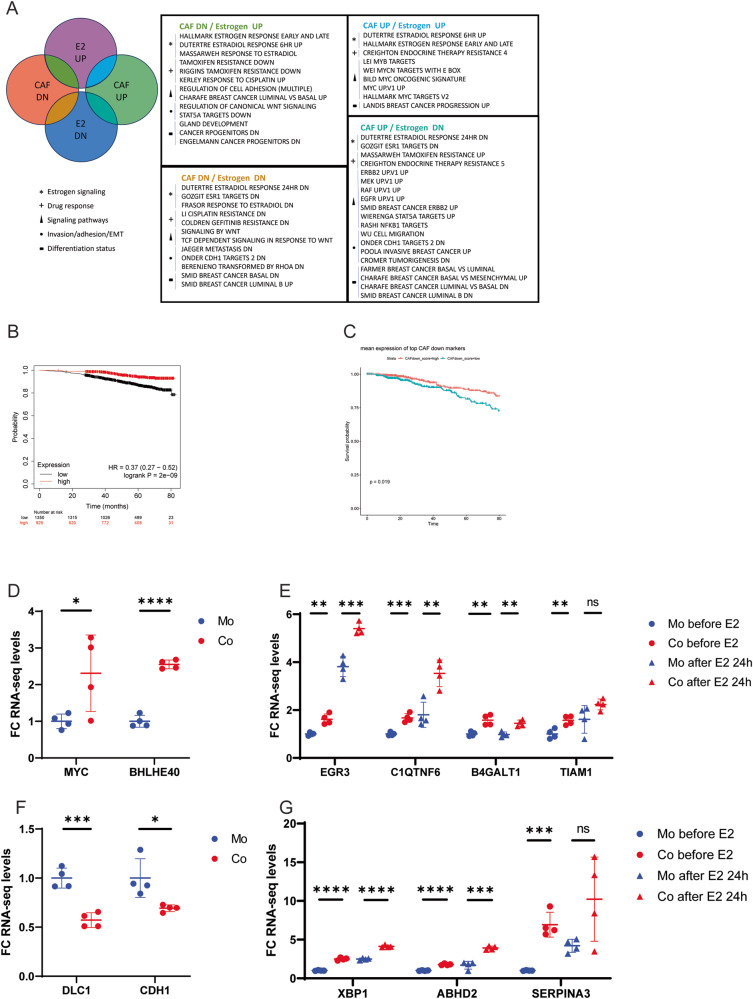
CAFs selectively modulate estrogen signaling. Metascape analysis of pathway enrichment based on RNA-sequencing of MCF7 cells subjected to mono- or co-culturing with CAF2 cells in the presence or absence of E2 (**A**). The pathways listed are statistically significant and selected representative examples from the results of analysis after either 6 h or 24 h of E2 stimulation (for full list of enriched pathways, see STables 3 and 4). A 94-gene signature composed of the genes downregulated by CAF2 in MCF7 cells by > 1.5 fold change served as a predictive biomarker for poor response to endocrine treatment in patients with ER-α^+^ tumors (*n* = 2279) [67] (**B**). Similarly, the predictive capacity of the gene signature was validated in the TCGA dataset (**C**). Regulation of key individual genes using RNA-Seq-derived Transcripts Per Million (TPM) values in MCF7 cells derived from mono- or co-cultures with CAF2 cells (**D**, **F**), and with or without 24 h stimulation with E2 (**E**, **G**). Represented as relative levels of untreated mono-cultures and displaying the mean with standard deviation. E2= estrogen; Mo = mono-culture; Co = co-culture.

To understand whether paracrine signaling from CAF2 modulates the entire ER-α gene expression program, or cause a selective regulation, we cultured MCF7 cells in the presence or absence of CAF2 while stimulating them with estrogen for 6 h or 24 h (SFig. 3A, schematic). Following differential gene expression analysis of the MCF7 cell transcriptome, genes were categorized as up- or down-regulated by either estrogen stimulation, CAF2, or both, and the data were visualized in a scatter plot (SFig. 3B (24 h), SFig 3C (6 h)). Of those genes regulated by both estrogen and CAF2, regardless of timepoint, the majority were induced by estrogen but repressed by CAF2, similar to the ERE-based luciferase readout. Pathway enrichment analysis through Metascape demonstrated that the genes that were regulated in this manner were enriched in gene sets involved in induction of estrogen response or suppression of tamoxifen resistance (Fig. 5A). By downregulating genes that estrogen induced, CAFs also acted to reduce the response to cisplatin, provoked a shift away from a luminal towards a basal phenotype, and enhanced cancer progenitor cell characteristics (Fig. 5A). Unexpectedly, despite counteracting the effect of estrogen on most bona fide ER-α target genes in the co-cultures, CAF2 also served to maintain a subset of the estrogen transcriptome, both among the estrogen-induced and estrogen-repressed genes (Fig. 5A), demonstrating that CAF2 can mimic the effect of estrogen in the absence of the hormone. Genes that were induced by either estrogen or by CAF2 alone were enriched in categories referred to as endocrine therapy resistance, breast cancer progression, and Myc-driven oncogenesis. Conversely, both estrogen and CAF2 similarly repressed genes that are commonly downregulated in basal *vs* luminal gene programs, Wnt signaling, and during the metastatic process. (Fig. 5A). Moreover, CAF2-induced genes were enriched in categories involved in endocrine therapy resistance, metastasis, basal-like identity, and oncogenic signaling pathways, despite estrogen negatively regulating these same pathways in MCF7 cells (Fig. 5A). The ability of CAFs to regulate responsiveness to endocrine therapy was further supported by the fact that low expression of a gene signature composed of the most highly downregulated genes (by fold change, cutoff >1.5) by CAF2 in MCF7 cells served as a predictive biomarker for poor response to treatment in patients with ER-α^+^ tumors in two different patient cohorts (Fig. 5B, C), again indicating suppression of hormonal pathways in luminal cancers by CAFs.

At the individual gene level, CAF2 conferred a more aggressive phenotype to MCF7 cells independent of estrogen stimulation, as demonstrated by the upregulation of the *MYC* oncogene (Fig. 5D) and the pro-survival and pro-metastatic transcriptional regulator *BHLHE40* (Fig. 5D) [16]. Additionally, CAF2 were able to induce genes in MCF7 cells both before and after estrogen stimulation that are involved in proliferation, invasion, and metastasis, such as *EGR3* (Fig. 5E*)* [17]*, C1QTNF6* (Fig. 5E) [18], *B4GALT1* (Fig. 5E) [19] and *TIAM1* (Fig. 5E) [20]. On the other hand, CAF2 lowered the expression of the tumor- and metastasis-suppressor *DLC1* (Fig. 5F) [21], as well as *CDH1* in estrogen-unstimulated conditions, further indicative of induction of EMT (Fig. 5F) [22]. Strikingly, the gene regulation was complemented with CAF2 boosting the levels of genes related to drug resistance, in the absence of estrogen, and yet even further in the presence of estrogen, such as *XBP1* (Fig. 5G) [23], *ABHD2* (Fig. 5G) [24], and *SERPINA3* (Fig. 5G) [25]. Taken together, our transcriptional analysis demonstrates that paracrine signaling from CAFs lead to an increased aggressiveness of luminal breast cancer cells by inducing the expression of oncogenes instigating EMT and pro-metastatic signaling to aid proliferation, invasion and metastasis, respectively; CAFs also demonstrate their ability to maintain selected estrogen-induced target genes.

### High throughput screening identifies signaling pathways involved in paracrine signaling by CAF2 to luminal breast cancer cells

**Fig. 6:**
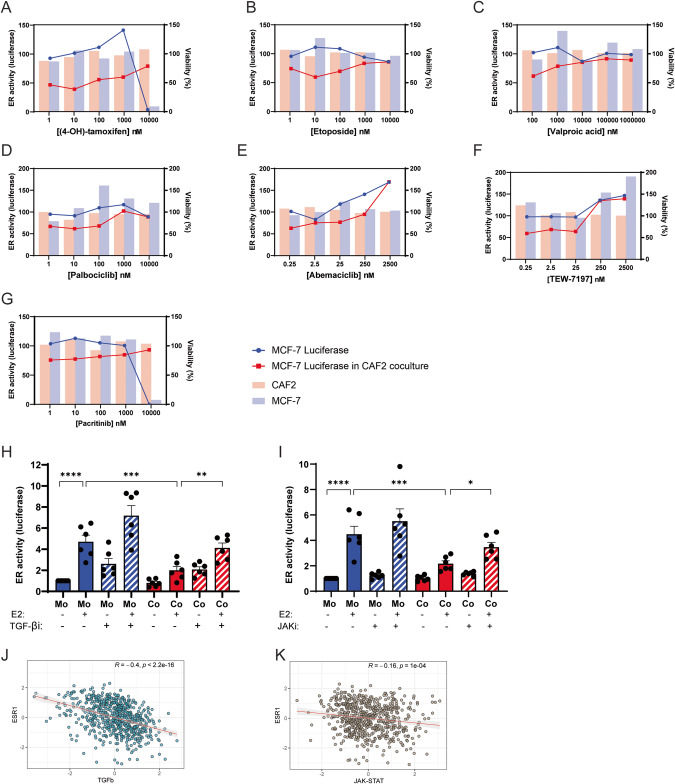
High throughput screening identifies signaling pathways involved in paracrine signaling by CAFs to luminal breast cancer cells. Examples of positive hits from the drug screen showing how the drug dose response affects MCF7 ERE-luciferase activity in mono- or co-cultures with CAF2 cells, as well as viability of both cell types in co-cultures. 4-OH Tamoxifen is shown as an example of a drug that reduce ERE activity (**A**). Drugs that abrogate the reduced luciferase activity induced by CAFs include the topoisomerase II inhibitor etoposide (**B**), the HDAC inhibitor valproic acid (**C**), the CDK4/6 inhibitors palbociclib (**D**) and abemaciclib (**E**), the TGF-β inhibitor TEW-7197 (**F**) and the JAK inhibitor pacritinib (**G**). The left y-axis displays the ER-α activity (as measured by luciferase) of MCF7 cells alone (blue line) or with CAF2 co-culture (red line). The bar graphs represent the viability of MCF7 cells (blue column), or the CAF2 (red column) on the right y-axis. The effect of TGF-β type I receptor inhibition and JAK inhibition was validated through the use of other inhibitors of the TGF-β type I receptor (SB-431542; TGF-βi, 5 µM) (**H**) or pan-JAK inhibitor Pyridone 6 (JAKi, 75 nM) (**I**) on CAF2-mediated reduction of ER-α-activity in MCF7 cells. Pathway activity scores derived from PROGENy demonstrate a significant negative correlation between TGF-β (**J**) and JAK (**K**) signaling pathway activity with the expression of *ESR1* in luminal breast cancers included in the TCGA dataset. Error bars: SEM with unpaired ordinary one-away ANOVA, Fisher’s LSD multiple comparisons test: *P < 0.05, **P < 0.01, ***P < 0.001, ****P < 0.0001.

In order to identify molecular effectors of the paracrine signaling from CAFs that impinge on ER-α activity in luminal breast cancer cells, we screened the Institute for Molecular Medicine Finland (FIMM) Oncology compound library composed of 528 approved (28%) or emerging oncology drugs (55%) or probes (17%) at five concentrations covering a 10,000-fold concentration range. We utilized the CAF2 co-culture system with the MCF7 ER-α reporter line in direct physical co-cultures (SFig. 4). Hits were identified if they raised the CAF2-inhibited ER-α activity of MCF7 cells to within 2 STD of the plate DMSO controls. Tamoxifen was used as a positive control, where ER-α activity and viability was lost in the MCF7 monocultures at high concentrations (Fig. 6A). Table 1 lists the 39 compounds that were found to counteract the paracrine suppression of ER-α-signaling in MCF7 cells by the CAF2 cell line, exhibiting a wide variety of inhibitory actions. Conventional chemotherapeutic drugs incorporating topoisomerase inhibition, such as etoposide, were abundantly represented (Table 1 and Fig. 6B), as well as multiple kinase inhibitors and HDAC inhibitors, *e.g*. valproic acid (Table 1, Fig. 6C). Interestingly, the CDK4/6 inhibitors palbociclib and abemaciclib, approved for treatment of hormone receptor-positive breast cancer, partially blocked the paracrine effect of CAF2 on MCF7 cells, indicating that combination treatment with CAF-targeting drugs may be beneficial (Table 1 and Fig. 6D, E). To validate the drug screen in an independent setting, we selected the TGF-β (TEW-7197, Fig. 6F) and the JAK (Pacritinib, Fig. 6G) signaling pathways, which were both represented among the hits. To block signaling from the TGF-β type I receptor ALK5, we incubated MCF7 cells either alone or in co-culture with CAF2 with the low molecular weight kinase inhibitor SB-431542. Indeed, whereas CAF2 reduced the ERE activity of MCF7 cells, this effect was abolished by the addition of SB-431542 (Fig. 6H). Similarly, CAF2 were unable to confer paracrine suppression of ER-α activity in the presence of the pan-JAK inhibitor Pyridone 6 (Fig. 6I), corroborating the results from the drug screen using the MCF7 cell line. The screen also indicated that TGF-β or JAK inhibition did not result in a direct suppression of growth in the CAF2 cell line (Fig. 6F, G), however in other experiments we noted that inhibition of these pathways caused a shift in CAF phenotype from α-smooth muscle actin-positive to -negative (SFig. 5A–H).

**Table 1:** Functional drug screening identified 39 candidate drug hits that interfered with the CAF2-inhibition of MCF7 ER-α activity at least one concentration included, without boosting MCF7 cell number or showing toxicity to the CAF2 cells.

	Drug name	Mechanism	Class
**1**	Chloroquine	Anti-malaria agent; chemo/radio-sensitizer	Chemotherapy
**2**	Daunorubicine	Topoisomerase II inhibitor	
**3**	Etoposide	Topoisomerase II inhibitor	
**4**	Teniposide	Topoisomerase II inhibitor	
**5**	Aldoxorubicin	Topoisomerase II inhibitor	
**6**	BMS863233	Cdc7 inhibitor	Kinase inhibitor
**7**	Abemaciclib	CDK4/6 inhibitor	
**8**	Palbociclib	CDK4/6 inhibitor	
**9**	PF-670462	CK1delta/CK1epsilon inhibitor	
**10**	DEL-22379	ERK dimerization inhibitor	
**11**	Ulixertinib	ERK inhibitor	
**12**	AMG-925	FLT3/CDK4 inhibitor	
**13**	Pacritinib	JAK2/FLT3 inhibitor	
**14**	Ruxolitinib	JAK1/2 inhibitor	
**15**	Peficitinib	JAK3 inhibitor	
**16**	UNC2881	MER inhibitor	
**17**	Foretinib	MET/VEGFR2 inhibitor	
**18**	Altiratinib	MET/Tie2 inhibitor	
**19**	LY-2584702	P70S6K inhibitor	
**20**	PF-00477736	P70S6K inhibitor	
**21**	LY3009120	pan-RAF inhibitor	
**22**	GDC-0084	PI3K/mTOR inhibitor	
**23**	Enzastaurin	PKCbeta inhibitor	
**24**	Ripasudil	ROCK inhibitor	
**25**	Hydroxyfasudil	ROCK/PKA/PKG/PRK inhibitor	
**26**	GSK269962	ROCK1/2 inhibitor	
**27**	TEW-7197	TGF-β type I receptor ALK4/5 inhibitor	
**28**	OTS-964	TOPK inhibitor	
**29**	Brivanib	VEGFR inhibitor	
**30**	UNC0642	G9a/GLP inhibitor	Differentiating and/or epigenetic modifier
**31**	Valproic acid	HDAC inhibitor	
**32**	Vidofludimus	DHODH inhibitor	
**33**	Celecoxib	COX-2 inhibitor	NSAID
**34**	Glasdegib	Smo inhibitor	Hedgehog inhibitor
**35**	Digoxin	Cardiac glycoside	Other
**36**	MK-0752	Gamma-secretase/Notch1 inhibitor	
**37**	E7820	Integrin-α2 expression inhibitor	
**38**	NVP-LGK974	PORCN inhibitor	
**39**	Varespladib	Secretory phospholipase A2 inhibitor	

To test the generality of these findings we first repeated the co-culture experiment in the presence of TGF-β or JAK inhibitors using other ER-α^+^ cell lines. Interestingly, the suppressive effect of CAFs on ERE activity in T47D cells exhibited partial sensitivity towards JAK pathway inhibition, but not towards TGF-β pathway inhibition (SFig. 6A, B), whereas the converse was true for BT474 Cl5 cells that restored ER pathway activity in the presence of TGF-β inhibition (SFig. 6C, D). Thus, yet other signaling events emanating from CAFs may contribute to the suppression of ER activity. To find further support for TGF-β and JAK as modulators of ER pathway signaling in human breast cancer, we analyzed the transcriptional profile of all tumors classified as Luminal A or B in the BRCA dataset in TCGA. We used the PROGENy tool to estimate TGF-β and JAK pathway activity based on the gene expression data and correlated to expression of *ESR1*. In support of our experimental findings, both TGF-β and JAK pathway activity were significantly inversely correlated to the expression of *ESR1* (Fig. 6J, K). In addition, the patients with the 25% highest TGF-β pathway activity scores exhibited a significantly shorter progression-free interval compared to the patients with the 25% lowest scores, indicative of a reduced benefit from endocrine therapy (SFig. 7A); the JAK pathway activity score was not associated with endocrine therapy resistance in luminal breast cancer patients (SFig. 7B). It should be noted that both the TGF-β and the JAK signaling pathways are broadly acting in different cell types that may have opposing effects on malignant progression and drug sensitivity, and thus bulk RNA-sequencing constitutes a blunt tool to uncover such relationships.

Taken together, our pharmacological screen demonstrates the involvement of TGF-β and JAK signaling in the paracrine crosstalk between luminal breast cancer cells and CAFs, and identify potential points of intervention for future combinatorial treatment regimens.

## Discussion

Here, we have detailed a role for CAFs in regulating hormone receptor signaling in luminal breast cancers through paracrine interactions leading to a muted and modified estrogen response. In experimental tumors, the degree of stromal infiltration was inversely correlated with ER-α expression, indicative of a direct regulation. Indeed, the activity of ER-α response elements was significantly reduced in co-cultures of CAF2 and luminal breast cancer cells. In keeping with the net reduced activity of the ERE reporter in co-cultures of CAF2 and MCF7 cells, many established ER-α target genes, such as *PGR* and *XBP1*, that were upregulated by estrogen in monocultures, were suppressed by the presence of CAF2, even upon ER-α activation. Interestingly, whereas many established ER-α target genes were downregulated in the presence of CAF2, a subset of genes related to drug resistance, proliferation and invasion/metastasis was maintained by CAF2, demonstrating a selective effect of paracrine signaling in order to augment the aggressiveness and estrogen-independence of malignant cells. Indeed, low expression of a signature of genes suppressed by CAF2 in MCF7 cells was predictive of poor response to endocrine therapy in two large patient cohorts. Finally, a drug screen identified candidate effector pathways for the paracrine signaling between CAFs and malignant cells, suggesting an involvement of TGF-β and JAK signaling, and opportunities for pharmacological interventions.

Cancer-associated fibroblasts have emerged as key regulators of many of the hallmarks of cancer, including support of tumor initiation, progression, dissemination and drug resistance [26, 27]. However, other studies demonstrate a tumor-repressive effect of CAFs that restrict malignant growth through encapsulation by extra-cellular matrix [28]. The conflicting conclusions may be reconciled by the recent detailing of subsets of CAFs in breast cancer, including matrix-producing (mCAF) and peri-vascular subtypes (vCAF), through analyses at the single cell level [29–32]. Whereas the precise origin and function of each CAF subset remains to be conclusively determined, we have previously demonstrated a role for Platelet-derived growth factor (PDGF) receptor-α^+^ mCAFs in specifying the molecular subtype of breast cancer through a paracrine signaling loop involving expression of PDGF-CC by triple-negative breast cancer cells [10]. The ability of MCAM^+^ fibroblasts to modulate ER-α expression, sustain estrogen-dependent proliferation, and maintain sensitivity to tamoxifen was demonstrated previously [33]. Here, we have made use of three human CAF cell lines to detail a suppressive, but selective, effect on ER-α signaling in luminal breast cancers. Culturing conditions to maintain CAF subtypes for prolonged periods of times remain to be established, making conclusions on potential subset-specific effects on ER-α signaling difficult at present. The future use of spatial transcriptomics or multiplex immunostaining will enable a more precise appreciation of the relation between CAF subtypes and ER-α signaling activity in human luminal cancers.

Pioneering work has demonstrated the crucial importance of the mammary gland stroma and/or fibroblasts for the embryonic and post-natal development of the mammary gland [34]. Indeed, mesenchymal cells are needed both for induction of the embryonic mammary gland placode [35–37] as well as for ductal morphogenesis [38] in an estrogen-dependent manner. Interestingly, Sakakura et al. found that isolated mammary mesenchyme provoked mammary gland epithelium to undergo an atypical, compact ductal branching [39]. Here, we demonstrate that estrogen signaling in malignant breast epithelial cells was selectively modulated by CAFs. In general, CAF2 suppressed the regulation by estrogen of classical ER-α target genes, including *PGR*, *CXCL12* and *MYBL1* [12–15]. However, a subset of genes induced by estrogen in luminal cell monocultures exhibited a raised baseline expression in CAF2 co-cultures without estrogen, and even higher expression levels following estrogen stimulation. Such genes were often related to processes involved in migration, invasion, metastasis and drug resistance, providing an explanation for the growth support of CAFs observed in co-transplantation experiments. It may seem counter-intuitive that CAFs have previously been demonstrated to support ER-α-driven breast tumors, yet also result in a decrease in the driver ER-α itself. Here, we show that during CAF2-induced reduction in ER-α, certain pro-tumorigenic downstream targets of ER-α are maintained, or even enhanced by the CAFs. Whether the paracrine signaling by CAFs utilize the same promoter elements as ER-α to maintain the selective expression of such genes, or whether there are CAF-specific gene regulatory mechanisms at play, remains to be determined. The results from our chemical screen for effector pathways may serve as a starting point for such studies. We validated TGF-β signaling through the type I receptor ALK5 and JAK signaling as mechanistically involved in the suppression of ER-α-signaling in malignant cells. Our study could not distinguish whether these signaling pathways are active in the CAFs, in the malignant cells, or both. Nevertheless, JAK is known to act downstream of ALK5 in a Smad-independent manner [40–42], conceivably acting concertedly in the same paracrine signaling cascade. Intriguingly, a recent study of inflammatory breast cancer demonstrated a JAK2/STAT3 signaling axis that conferred chemoresistance and EMT, consistent with our findings of CAF2-regulated malignant cell traits [43]. Furthermore, CAF-modified genes, including B4GALT1 and BHLHE40, are highly correlated with, or induced by, TGF-β in breast cancers [44], and DLC1 expression, which was inhibited by CAFs in breast cancer cells, was shown previously to modify a subset of TGF-β-induced genes that promoted osteolytic bone metastasis via paracrine interactions [21]. A recent drug screen found Polo-like Kinase1 (PLK1) inhibitors as inducers of ER-α levels and activity, in studies performed on TNBC monocultures. [45] However, in our drug screen the three PLK1 inhibitors included were not candidates of CAF-induced reduction of ER-α activity, but instead were highly toxic to the luminal MCF7 cells.

We show that CAFs have a strong influence on ER-α-driven breast cancer cells in the short-term. Furthermore, our long-term co-cultures demonstrated that the ER-α expression was reduced but not entirely lost, even when the selective pressure from CAFs was removed, suggesting in part epigenetic regulation. Indeed, epigenetic modifiers, including valproic acid and UNC0642, were represented in our top hits from the chemical screen of compounds that prevented CAF2 from reducing ER-α activity in breast cancer cells. Strikingly, the triple-negative breast cancer phenotype has previously been demonstrated to be epigenetically regulated [46]. In this context, it may also be interesting to consider the effects of CAFs on the properties of the extra-cellular matrix, such as matrix density and stiffness. Future studies will have to determine whether mechano-transduction pathways may be involved in epigenetic reprogramming of breast cancer cells into a hormone-independent state.

Our co-transplantation studies demonstrate that CAFs confer an advantage to MCF7 tumor growth when estrogen levels are restricted, either by supporting survival of malignant cells, or by selecting for estrogen-independent tumor growth. The low estrogen exposure during this experiment simulates endocrine therapy of luminal breast tumors with *e.g*. selective ER-α modulators or aromatase inhibitors; CAFs may thus contribute to endocrine treatment resistance. Indeed, a gene signature composed of genes downregulated in MCF7 cells by CAF2 held predictive capacity of poor response to endocrine therapy in patients with luminal breast tumors. However, we did not see the ability of MCF7 cells to establish tumors when mice were left completely unexposed to exogenous estrogen, even in the presence of CAF2 [47]. Taken together, our work suggests an opportunity for combination treatment strategies targeting paracrine signaling from CAFs together with endocrine therapy for breast cancer. Several strategies involving clinically approved drugs may be envisioned: 1) Combination of CAF-targeting and endocrine therapy, given the reduced sensitivity to estrogen conferred by CAFs to luminal breast cancer cells; 2) Combination of CAF-targeting and conventional chemotherapy, motivated by the upregulation of genes involved in drug resistance by CAFs; 3) Combination of CAF-targeting and CDK4/6 inhibitors, given the abolished paracrine suppression of ER-α signaling by CAFs in the presence of palbociclib and abemaciclib; 4) CAF-targeting followed by endocrine therapy in TNBC patients, inspired by our previous work detailing a role for CAFs in maintaining the ER-α^-^ phenotype [10]. Indeed, we are currently conducting a clinical phase 2 trial with a window-of-opportunity design in which TNBC patients are treated with the CAF-targeting PDGF-receptor inhibitor imatinib during the time between diagnosis and surgery, with frequency of ER-α^+^ malignant cells as the primary endpoint (ClinicalTrials.gov Identifier: NCT05722795). Similarly, HDAC inhibitors have been demonstrated to convert TNBC cells to ER-α^+^ through epigenetic reprogramming [46, 48]. Interestingly, the HDAC inhibitor valproic acid also reduced the effect of paracrine CAF2 signaling in our co-culture screen, indicating that epigenetic marks may also regulate the ability of CAF2 to support hormone-independence in breast cancer cells. In addition, our study identifies combinations of endocrine therapy with TGF-β inhibition or JAK inhibition as treatment regimens warranting further studies.

Only by improving our understanding of the organizational principles of the tumor microenvironment, and the prognostic and predictive implications of functional niches composed of defined subsets of stromal cells and malignant cells, will we be able to develop tools for precision medicine for cancer. The recent surge of studies detailing stromal heterogeneity at the single cell level must now be followed by a reciprocal rush of functionally informative studies detailing the network of paracrine crosstalk with equally high resolution. As demonstrated by our present study, such microenvironmental conversations are instructive for the management of malignant diseases, including breast cancer, and may hold the key for developing rationally designed combination therapies.

## Methods

### Cells and reagents

MCF7 (#HTBB-22), T47D (#HTB-133), and BT-474 clone 5 (Herceptin resistant, #CRL-3247) were obtained from ATCC. The CAF2 cell line are immortalized human-derived breast fibroblasts primed by MCF7 co-implantation xenografts as described by Polanska et al. [11] and were obtained from Akira Orimo, Juntendo University, Japan. hTERT-immortalized CAFs-A and -B were isolated from a breast carcinoma and were a gift from Ole W. Petersen and Lone Rønnov-Jessen, University of Copenhagen, Denmark [49]. T47D and CAF2 cells were cultured in DMEM (Corning #10-013), BT-474 cultured in RPMI-1640 (Corning #10-040) and MCF7 in RPMI-1640 (Corning #10-040) supplemented with sodium pyruvate 1 mM (Corning #25-000-CIR). CAF2-primed Long-term (LT) MCF7 cultures were generated by serially culturing MCF7 parental line in transwells containing DMEM (Corning #10-013) with CAF2 cells below for 50 passages, or alone for the same number of passages in transwells. All growth media contained 10% FBS (Corning #35-010-CV) and 1% Penicillin, Streptomycin (Corning #30-001-CI) and cells were maintained in 21% O_2_ and 5% CO_2_, 37 ^o^C. Cells were regularly tested for mycoplasma infection. For ER-α-activity and co-culture experiments, both starvation and experimental media were Phenol-red free DMEM (PRFDM, Gibco #31053028), 5% charcoal stripped serum (Gibco #12676029) with Penicillin and Streptomycin (Corning #30-001-CI). SB-431542 (Tocris #1614) and Pyridone-6 (Tocris #6577) were dissolved in DMSO and used at final concentrations of 5 µM and 75 nM respectively. 17-Beta-estradiol (referred to as estrogen in the text (E2) (Sigma-Aldrich #E2758) was dissolved in ethanol and used at final concentrations of 100 nM.

### Luciferase assay

Cells were stably infected with a lentivirus expressing luciferase under the control of 3 consecutive Estrogen Receptor Elements (ERE) generating ER-α activity reporter lines. Cells were treated and lysed in passive lysis buffer (#E1941, Promega) and the supernatant analyzed for luciferase activity using a luciferase assay (#E1500, Promega). Luciferin substrate was added, and luminescence was quantified using the Synergy II microplate reader. In the high throughput drug screen ONE-Glo (Promega #E6120) was added directly to the medium before reading the luminescence.

### Conditioned medium collection

Breast cancer cells were seeded in growth media overnight before being washed in PBS and then starved of estrogen using PRFDM for 24 h. Conditioned media (CM) of the CAF2 was collected following 48 h in PRFDM and concentrated in Amicon filters with a molecular weight cut-off of 10 kDa. Cell culture medium conditioned by an approximate surface area of 25–50 cm^2^ of near-confluent CAF2 was concentrated and divided per 0.4 × 10^6^ cancer cells in a well of a 6-well plate for 48 h in the absence or presence of 100 nM estradiol before lysis.

### Transwell cocultures

Cancer cells were seeded into polyester transwells (0.4 µm pore size, #734–1577, VWR or #9300412, cellQART) and CAF2 seeded in separate 6-well plates in a 1:3 ratio in their respective culture media overnight. Cancer cells were starved for 24 h in PRFDM and maintained throughout experiments. Transwells containing starved cancer cells were transferred to plates containing CAF2 and then maintained in PRFDM for 48 h with or without Estradiol (100 nM) before lysis. A polystyrene scraper was used to gently remove lysates from wells or transwells.

### Western blotting

MCF7 (in mono- and co-culture with CAF2 at a ratio of 1:3) and CAF2 were seeded in 6 well plates for 4 days. Cells were then lysed in RIPA buffer (ThermoFisher #89901). BCA (ThermoFisher #23227) assay was used to determine protein content and load 30 µg of lysate into a Mini-PROTEAN TGX gel (Bio-Rad #4561093). Proteins were then transferred into a PVDF membrane (Bio-Rad #1704156) with Trans-Blot Turbo Transfer System. The membrane was incubated for 1 h in 5% milk in TBST and then incubated over night with Estrogen Receptor Beta monoclonal antibody (Invitrogen #MA5–24807; 1:1000), followed by secondary antibody (Cell Signaling #7076; 1:2000) incubation for 1 h. After 15 min incubation with stripping buffer (ThermoFisher #46430), membrane was re-blocked in 5% milk in TBST and incubated overnight with Anti-Estrogen Receptor alpha (Abcam #ab3575; 1:1000) antibody. Secondary antibody (Cell Signaling #7074; 1:2000) and primary anti-GAPDH HRP-linked antibody (Cell Signaling #3683; 1:3000) were incubated for 1 h and 30 min, respectively. Blots were incubated for 3 min with HRP substrate (Millipore #WBLUF0500) and chemiluminescence was detected with the Amersham Imager 600 (Cytiva). Band intensities were quantified with the Image Lab software (Bio-Rad) and plotted with GraphPad Prism 9.3.1 (GraphPad Software).

### Proliferation assay

MCF7 and CAF2 were seeded in 6 well plates at a ratio of 1:3 in DMEM/F-12 (ThermoFisher #11330032), 10% FBS (Corning #35–010-CV), 1% Penicillin and Streptomycin (Corning #30–001-CI) and DMEM (Corning #10-013) containing 10% FBS (Corning #35-010-CV) and 1% Penicillin, Streptomycin (Corning #30-001-CI), respectively. Treatment of MCF7 with estradiol (100 nM and 10 nM) was carried out after 24 h of starvation in DMEM/F-12 (ThermoFisher #11330032) containing 5% charcoal stripped serum (#12676029 Gibco) and 1% Penicillin and Streptomycin (Corning #30-001-CI). Transwells containing CAF2 were moved into MCF7 containing plates the following day. Proliferation rate was tested after 3 days of treatment.

To test the effect of JAK and TGFβ inhibition alone and in combination with 4-Hydroxytamoxifen, MCF7 were seeded in 6 well plates in DMEM/F-12 (ThermoFisher #11330032), 10% FBS (Corning #35-010-CV), 1% Penicillin and Streptomycin (Corning #30-001-CI). 4-Hydroxytamoxifen (Sigma-Aldrich #SML1666), SB-431542 (Tocris #1614) and Pyridone-6 (Tocris #6577) were added every other day for 5 days at final concentrations of 1 µM, 5 µM and 75 nM, respectively, Proliferation rate was tested after 5 days of treatment.

To test proliferation rate, the SRB assay (#S9012-56 Sigma) was performed following the protocol in [50]. Absorbance was quantified using the Synergy II microplate reader.

### Reverse transcription and quantitative PCR

Cells were lysed in RLT buffer and passed through a QiaShredder (Qiagen #79656) before RNA was isolated using the RNeasy kit (Qiagen #74106). One microgram of RNA was used in the cDNA synthesis reaction using the iScript kit (Bio-Rad #1708891). Quantitative PCR was performed using SYBR-green (Thermo # 4364344) and mRNA expression normalized to the housekeeping gene RPL19 or both RPL19/HPRT. Primer sequences used were as follows:

*PR* F: GAGCTTAATGGTGTTTGGTC, R: GTTTGACTTCGTAGCCCTT

*ERBB2* F: CCCATCTGCACCATTGATGTC, R: GAGTCAATCATCCAACATTTGACC

*CXCL12* F: ATTCTTCGAAAGCCATGTTGC, R: TTTCTCCAGGTACTCCTGAATCC

*MYBL1* F: AGGCAAGCAGTGTAGAGAAAGA, R: CGATTTCCCAACCGCTTATGT

*ESR1* F: GATCAACTGGGCGAAGAG, R: GATCTCTAGCCAGGCACATT

*RPL19* F: AAACAAGCGGATTCTCATGG, R: GCGTGCTTCCTTGGTCTTAG

*HPRT* F: ACCACCGTGTGTTAGAAAAGT, R: GGGAACTGCTGACAAAGATTCAC

### RNA-seq data processing and analysis

MCF7 cells were seeded in Transwells and CAF2 in 6-well plates in a 1:4 ratio in growth medium overnight. MCF7 cells were starved for 24 h before adding transwells to the CAF2 also in PRFDM for a further 2 days. Cultures were stimulated with 100 nM estradiol at 6 and 24 h before all being lysed in RLT at the same endpoint. RNA was isolated as described above and RNA sequencing and processing was performed by the Center for Translational Genomics, Lund University. Briefly, the raw data was generated using NextSeq 500 (SY-415-1001, Illumina). Demultiplexing raw data to FASTQ files was performed using bcl2fastq (Illumina) followed by a quality assessment of the FASTQ files using FastQC [51]. HISAT2 [52] was used to align the reads to the human reference genome GRCh38. Reference genome and annotation (GTF file) were downloaded from the Ensemble database release 94 [53]. StringTie [54] was used for the assembly of full transcripts and quantification of the expression levels at the gene and transcript level [55]. Default settings were used for all tools, unless otherwise specified. Transcriptomic analysis was performed in R [56]. Differential gene expression analysis was performed using Deseq2 (v1.26) [57] to assess the effect of CAF2, or estrogen treatment, at 6 and 24 h separately. For this we used a model with interaction: ~ cells + treatment + cells*treatment, where treatment = E2 or no E2, and cells = noCAF2 or CAF2. We used the *results* function with the *contrast* argument followed by the *LfcShrink* function, using type = “ashr” [58]. The effect of CAF was defined by comparing CAF2 vs no CAF2 (ctl) in the absence of estrogen (no E2 condition), while the effect of E2 was defined by comparing E2 vs no E2 in the absence of CAF2 cells (MCF7 cells alone). All Deseq2 results are shown in STable 1 (6 h E2) and STable 2 (24 h E2).

### Analysis of RNAseq data from the TCGA database

TCGA breast cancer RNA expression and clinical data [59, 60] were downloaded using the cgdsr package (version 1.3.0) provided by the cBioportal database [61, 62]. Based on the available PAM50 molecular subtype information, 420 Luminal A and 194 Luminal B tumors were selected for further analysis. The Surv() and survfit() functions in the survival package (version 3.5–5 [63]) was utilized for the survival analysis and the Kaplan–Meier survival curves were drawn using the ggsurvplot() function in survminer (version 0.4.9 [64]). Survminer’s log-rank test was used to test the statistical difference in survival rate between the groups.

We used PROGENy (version 1.16.0, [65]) to infer signaling activities of pathways in the luminal cases based on the top 500 most responsive genes as recommended by the PROGENy developers. The Pearson correlation coefficients with p-values were calculated using ggpubr (version 0.6.0, [66]).

### Kaplan-Meier analysis

Gene IDs for genes downregulated with a fold change > 0.5 in MCF7 cells in co-culture with CAF2 cells, compared to MCF7 mono-cultures, were used as input to the Breast RNA-seq dataset at kmplot.com [67]. Patients with ER-α^+^ tumors were dichotomized according to the median of the mean expression of the gene signature.

### Pathway enrichment analyses

Overrepresentation analyses for each gene list were performed using Metascape [68]. The ontology catalogs selected were GO biological process, Hallmark, BioCarta and Reactome gene sets, KEGG and pathways, CORUM complexes, chemical and genetic pertirbations, oncogenic signatures, immunologic signatures and canonical pathways from MSigDB, TRRUST and Transcription factor targets. We defined the background gene list as all genes for which DGE testing was performed.

### Drug library screening

The FIMM compound library was printed on collagen-coated plates (#152041) resuspended in 25 nl of DMSO and kept in a protective atmosphere until use (airtight storage, reduced humidity and oxygen). MCF7 cells were seeded alone or in coculture with CAF2 in a 1:3 ratio (3300:10000 cells) to a total volume of 25 ul PRFDM in 8 × 384 Well plates each, covering the drug library. After 48 h, Estradiol was added in 2 ul to each well for a further 48 h. TMRM (tetramethylrhodamine, methyl ester) was added to the wells and used as a viability marker along with NucBlue staining (ThermoScientific). Cells were imaged following 1 h incubation using the Opera Phenix™ High Content Screening System (Perkin Elmer). Together with the inherent GFP expression from the CAF2 cell line, the cell types could be distinguished, viability was assessed in the monocultures (MCF7) and the cocultures (CAF2) following drug exposure. The luciferase activity of MCF7 cells was assessed by adding ONE-Glo (Promega #E6120) to the medium. Chemiluminescence was recorded with a multimodal plate reader (EnSight, PerkinElmer). Raw luminescence readings were merged with compound and concentration information using an in-house R-script and curve-fitting was run using the Breeze (breeze.fimm.fi) pipeline. This provides screen quality controls metrics, drug response curves and drug sensitivity score (DSS), a modified area under the curve parameter described previously [69]. Drug compound library hits were identified if they passed the following criteria: increased the CAF2-inhibited ER-α levels to within 2 STD of the plate DMSO controls and did not cause high toxicity in either cell line ( > 40%) nor alter ER-α activity in the monocultures by more than 40%, which would suggest an effect regardless of CAF2 presence.

### Animal studies

The MCF7 xenografts utilized 37 female NSG (NOD.Cg-Prkdcscid Il2rgtm1Wjl/SzJ) from JAX (#5557) over all experiments. Pellets containing 5-fold decreasing amount of 17β-Estradiol with a 60-day release time: 0.5 mg, 0.1 mg, 0.025 mg (Innovative Research of America #SE-121) were inserted into 7-week old mice one day before tumor cell implantation. Pellets were inserted using a sterile trochar at the back of the neck whilst under anesthesia. Human MCF7 cells (0.5 × 10^6^) were implanted either alone or together with human CAF2 cells (1.5 × 10^6^) in a 1:3 ratio in 100 µl PBS in the 4th mammary fat pad. Tumors were measured 1–2 times per week with an electronic caliper. Tumor volumes were estimated using the formula: (L x W x W x π)/6. Mice were perfused in the heart under terminal anesthesia with PBS.

### Tissue preparation, histology and immunostaining

Tumors were fixed in Zinc Formalin fixative overnight and stored in ethanol before dehydration and paraffin embedding. Five micrometer-thick sections were deparaffinized and rehydrated before an antigen retrieval step (pH9 Dako #S2367) using a pressure cooker (2100 retriever, Prestige Medical). Additional endogenous peroxidase quenching was carried out with Bloxall (Vector Laboratories #SP-6000) for 20 min, followed by saturation of unspecific sites with CAS block (ThermoScientific #8120) for 30 min. ER-α was stained using the Rabbit anti-human SP1 clone (ThermoScientific #RM9101, 1:200) in a DAKO cytomation Autostainer plus followed by a Rabbit Envision secondary antibody incubation (#K400311-2 from Agilent). Slides were stained with HTX and Eosin (3 min), washed in water and dehydrated before mounting and imaging. Full-tissue images were acquired with a NanoZoomer S60 digital slide scanner, followed by visualization for annotation and analysis with the NDP.view2 software (both from Hamamatsu Photonics, Japan).

### Image analysis

Positive cell detection on the hematoxylin channel was performed in QuPath (v0.4.3) [70] to cell segment tumors. Subsequently, a trained object classifier identified cells expressing or not ER-α, and the resulting classification served as input to generate density maps with a radius of 100 µm. The *createAnnotationsFromDensityMap* function was then utilized to annotate areas with distinct stromal contents. Annotations with a density of ERα+ cells equal to or greater than 70% were categorized as low stromal regions, those between 26% and 69% as medium stromal regions, and those exceeding 25% as high stromal regions. DAB optical density was measured in QuPath (v0.4.3) [70] and visualized in R using ggplot2 [71] and ggpubr [66].

### Immunofluorescence staining

CAF2 were seeded in 6 well plates with or without transwells containing MCF7 in a ratio of 3:1. Treatment with SB-431542 (Tocris #1614) and Pyridone-6 (Tocris #6577) at final concentrations of 5 µM and 75 nM, respectively, was performed in DMEM (Corning #10-013) containing 10% FBS (Corning #35-010-CV) and 1% Penicillin, Streptomycin (Corning #30-001-CI) and cells were maintained in 21% O_2_ and 5% CO_2_, 37 ^o^C. Cells were rinsed with PBS and fixed with cold methanol for 5 min at −20 °C. Wells were incubated for 5 min with protein block solution (Dako #X0909) and anti-αSMA antibody (Sigma #A2547; 1:1000) was incubated for 1 h at room temperature. Secondary antibody (Invitrogen #A21203; 1:500) incubation was carried out for 30 min at room temperature and wells were counterstained with DAPI (Invitrogen #D3571; 1:3000). Images were acquired with an automated BX63 microscope connected to a DP-80 camera (Olympus). Positive cell detection on the DAPI channel was run in QuPath with an intensity threshold of 10 and a cell expansion of 15 µm. A threshold classifier was then run on αSMA channel (cutoff = 48) and intensities were plotted with GraphPad Prism 9.3.1 (GraphPad Software).

### Statistical analysis

Statistical analysis of data from in vitro experiments was performed on at least three biological independent replicates, taking a mean of the technical replicates measured. Analysis of the IHC staining quantification was performed using the unpaired two-tailed t-test. Luciferase assays and qPCR were analyzed using the unpaired ordinary one-away ANOVA with Fisher’s LSD multiple comparisons test. In vivo tumor growth was analyzed by multiple unpaired t-tests (Two-stage step-up according to Benjamini, Krieger, and Yekutieli) with an FDR of 1%. Kaplan-Meier analyses were statistically queried with the log-rank test.

### Study approval

All animal experiments were approved by the ethical committee for animal experimentation under the Lund University ethical permit M167-15 and 14122/2020.

### Supplementary information

SFig 1

SFig 2

SFig 3A-B

SFig 3C

SFig 4

SFig 5

SFig 6

SFig 7

Supplemental figure legends

STable 1

STable 2

STable 3

STable 4

## Data Availability

All sequencing data are deposited and freely available in the NCBI GEO database, accession # GSE251644.
