# Supplementary figures and images for: Cancer-associated fibroblasts rewire the estrogen receptor response in luminal breast cancer, enabling estrogen independence
Source: Oncogene. 2024 Feb 22;43(15):1113–26. doi: 10.1038/s41388-024-02973-x (PMC10997519; doi:10.1038/s41388-024-02973-x)

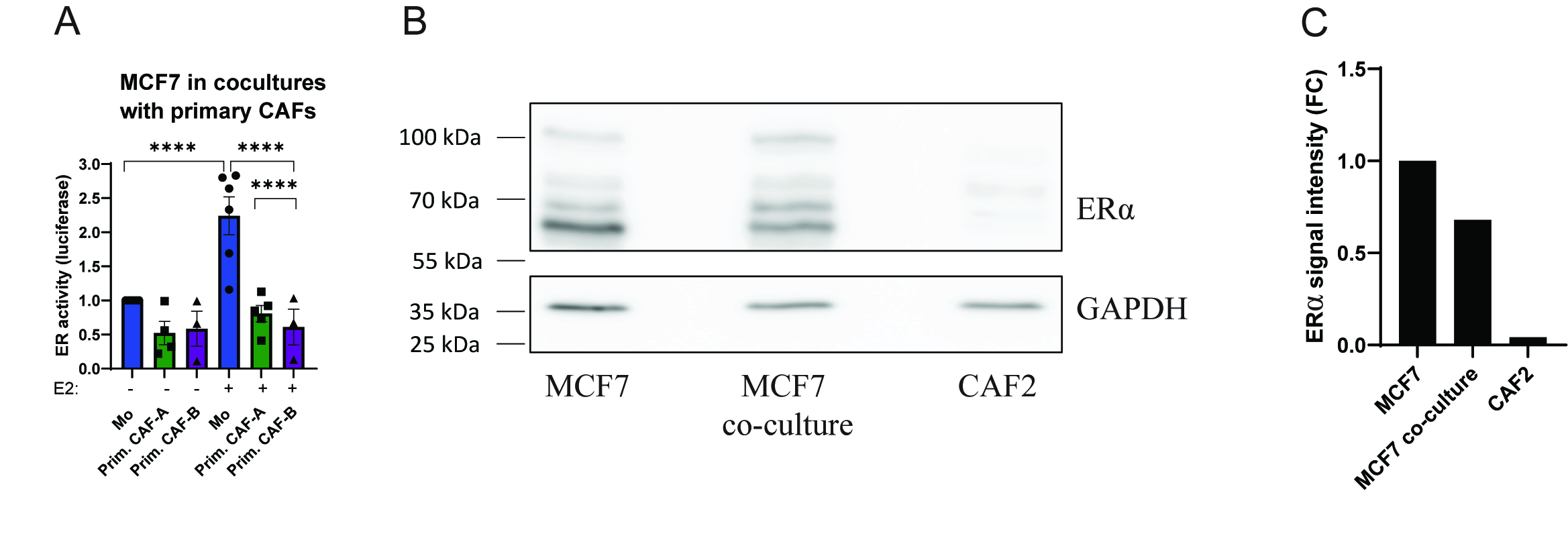

Supplement: Supplementary file 1 — SFig 1 [file 41388_2024_2973_MOESM1_ESM.tif]

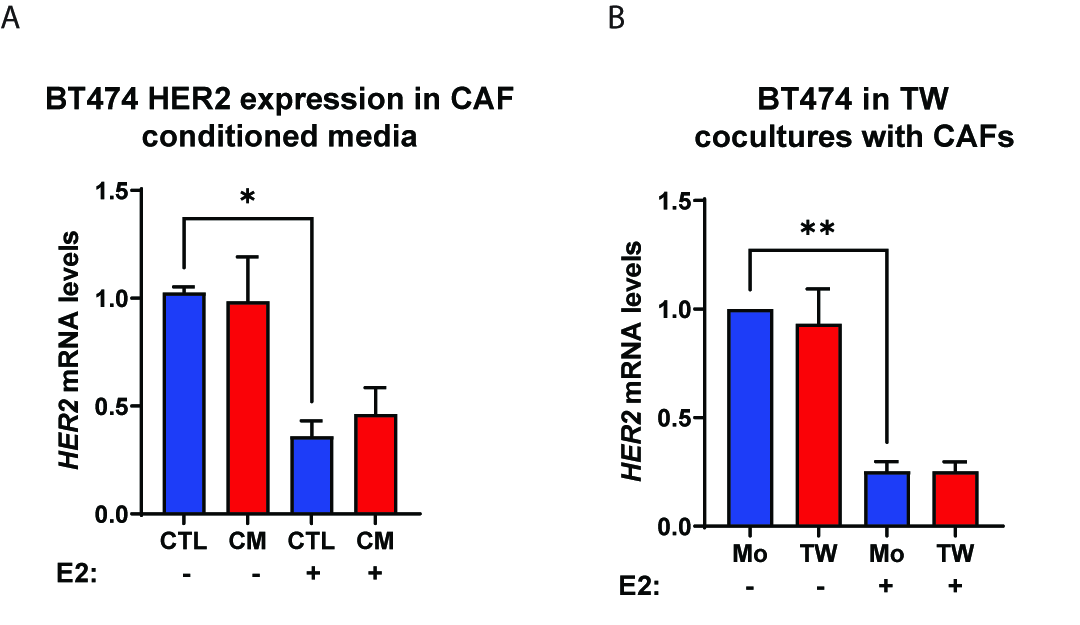

Supplement: Supplementary file 2 — SFig 2 [file 41388_2024_2973_MOESM2_ESM.tif]

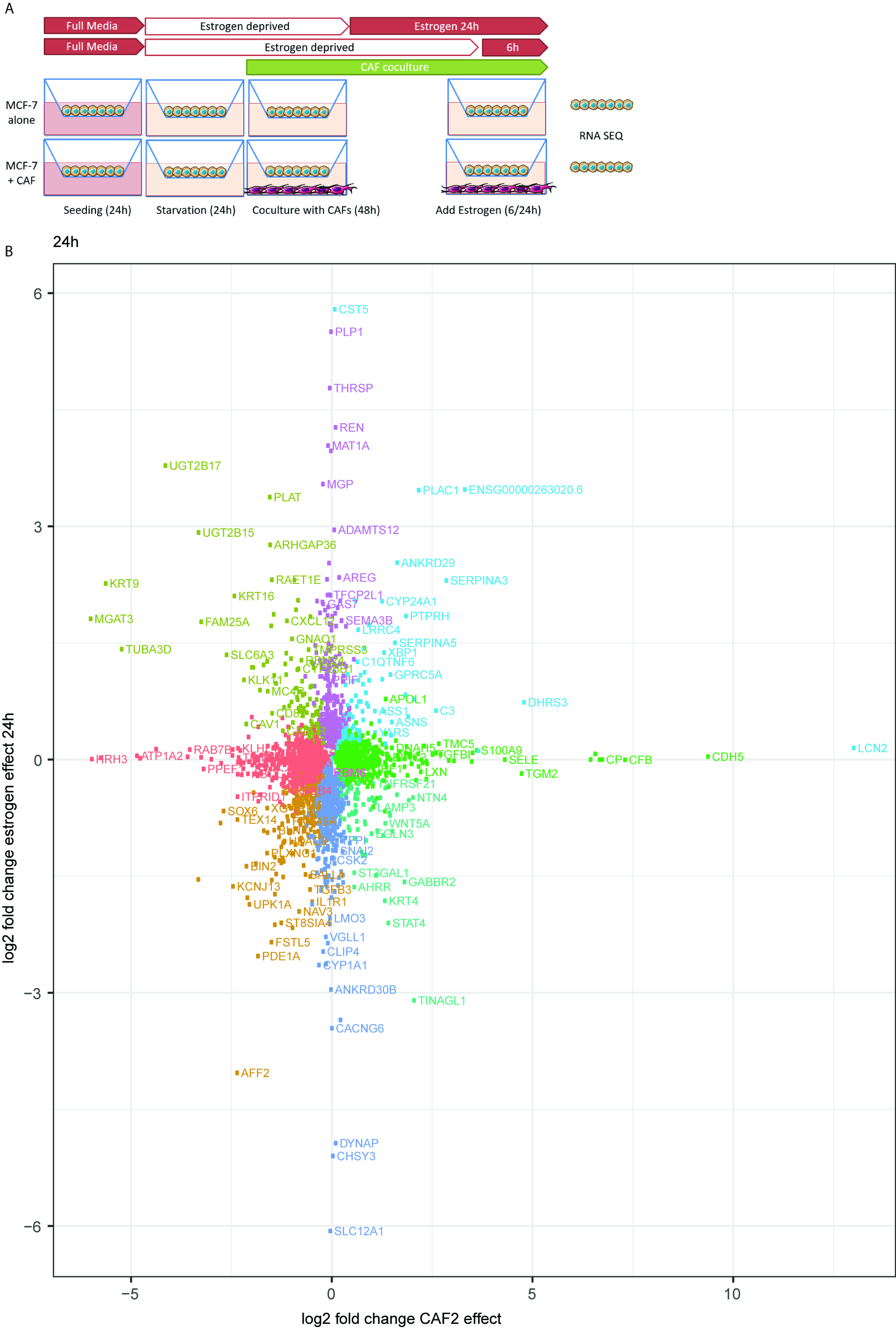

Supplement: Supplementary file 3 — SFig 3A-B [file 41388_2024_2973_MOESM3_ESM.tif]

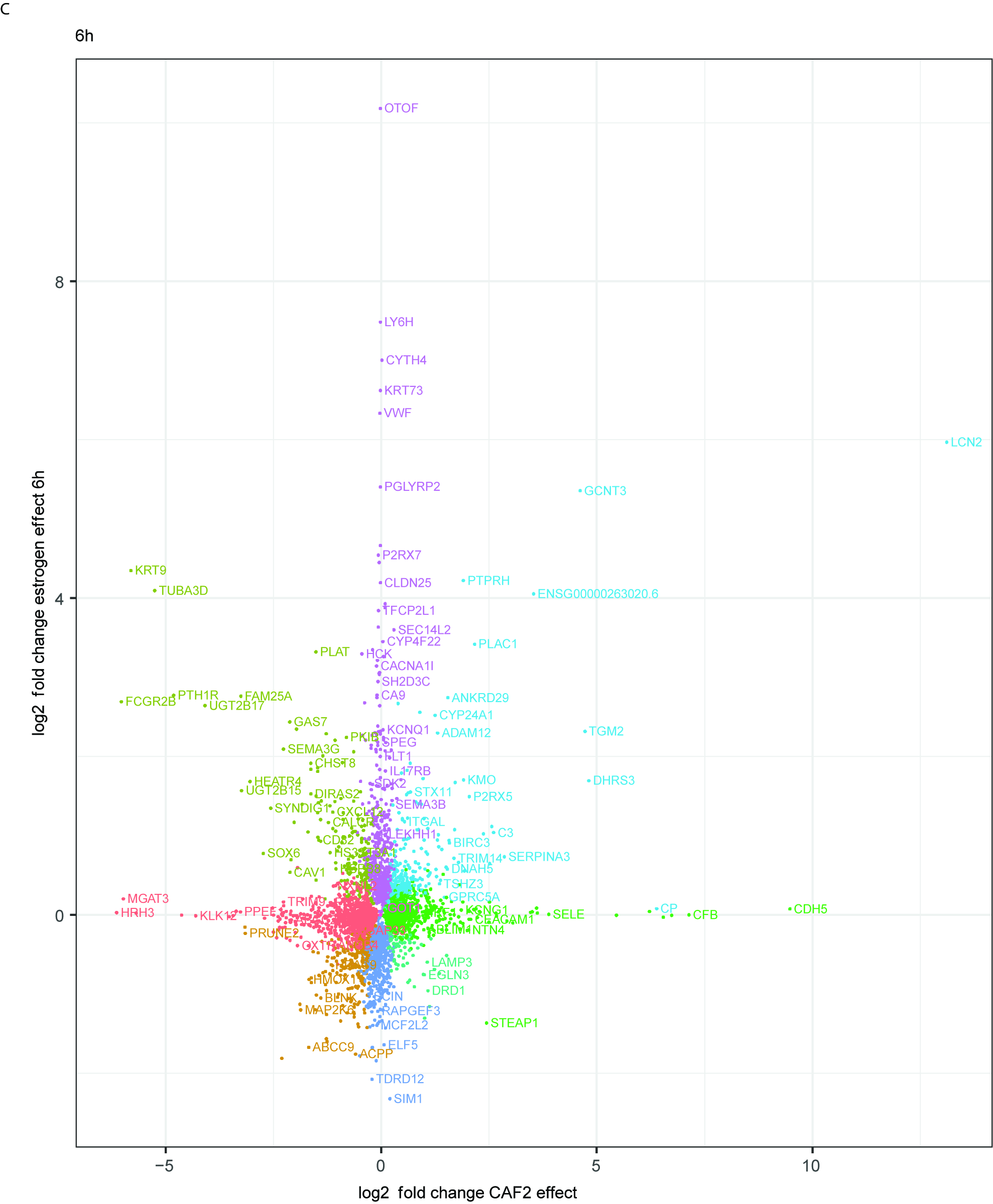

Supplement: Supplementary file 4 — SFig 3C [file 41388_2024_2973_MOESM4_ESM.tif]

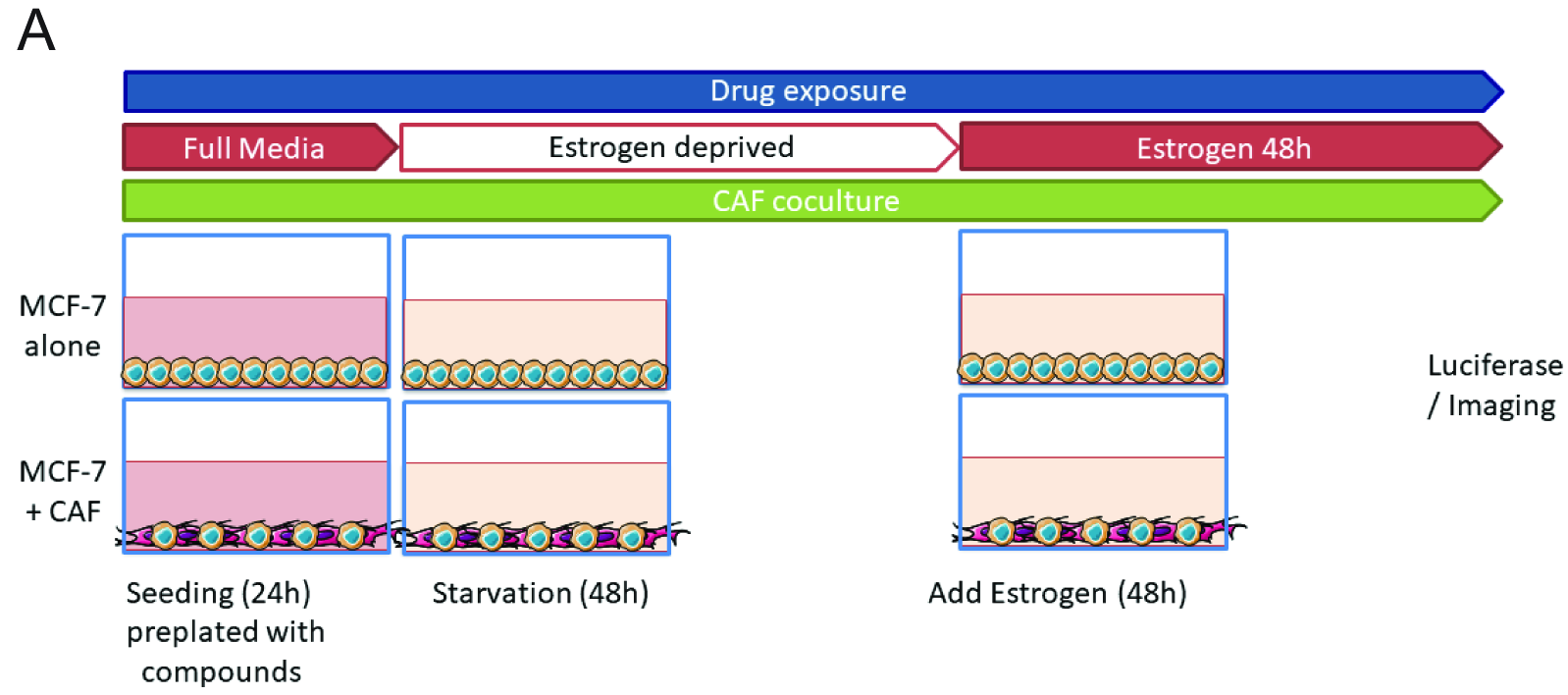

Supplement: Supplementary file 5 — SFig 4 [file 41388_2024_2973_MOESM5_ESM.tif]

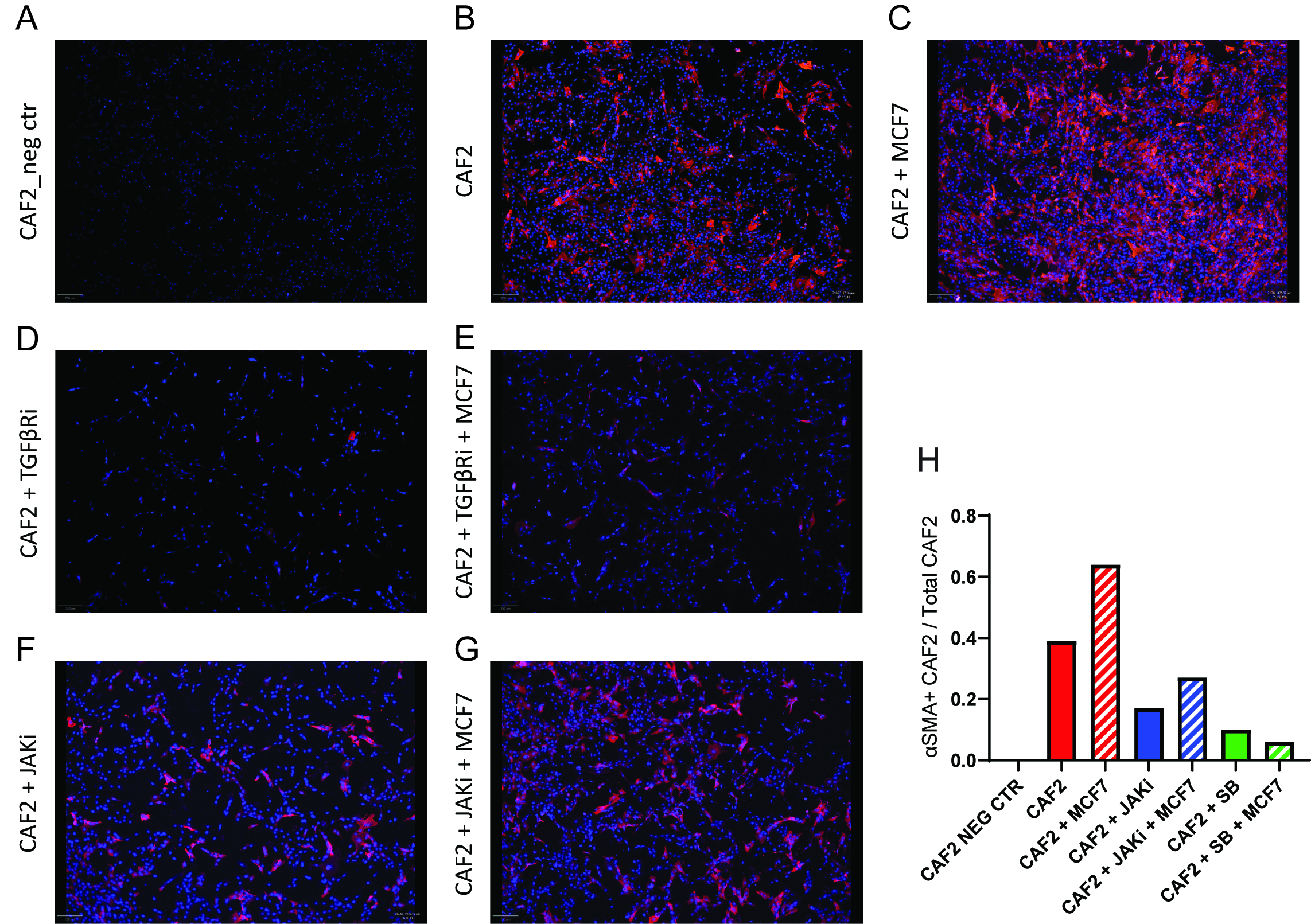

Supplement: Supplementary file 6 — SFig 5 [file 41388_2024_2973_MOESM6_ESM.tif]

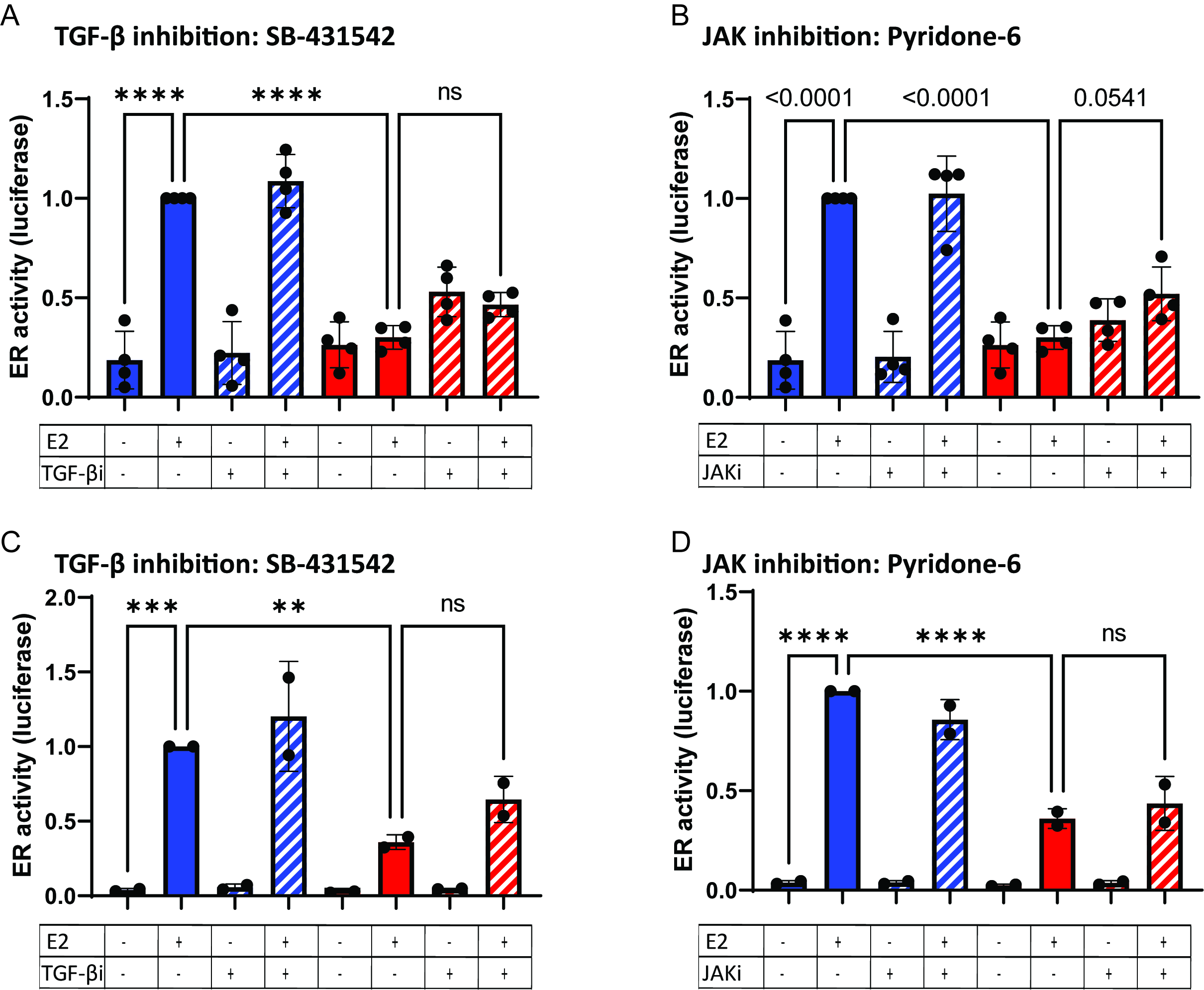

Supplement: Supplementary file 7 — SFig 6 [file 41388_2024_2973_MOESM7_ESM.tif]

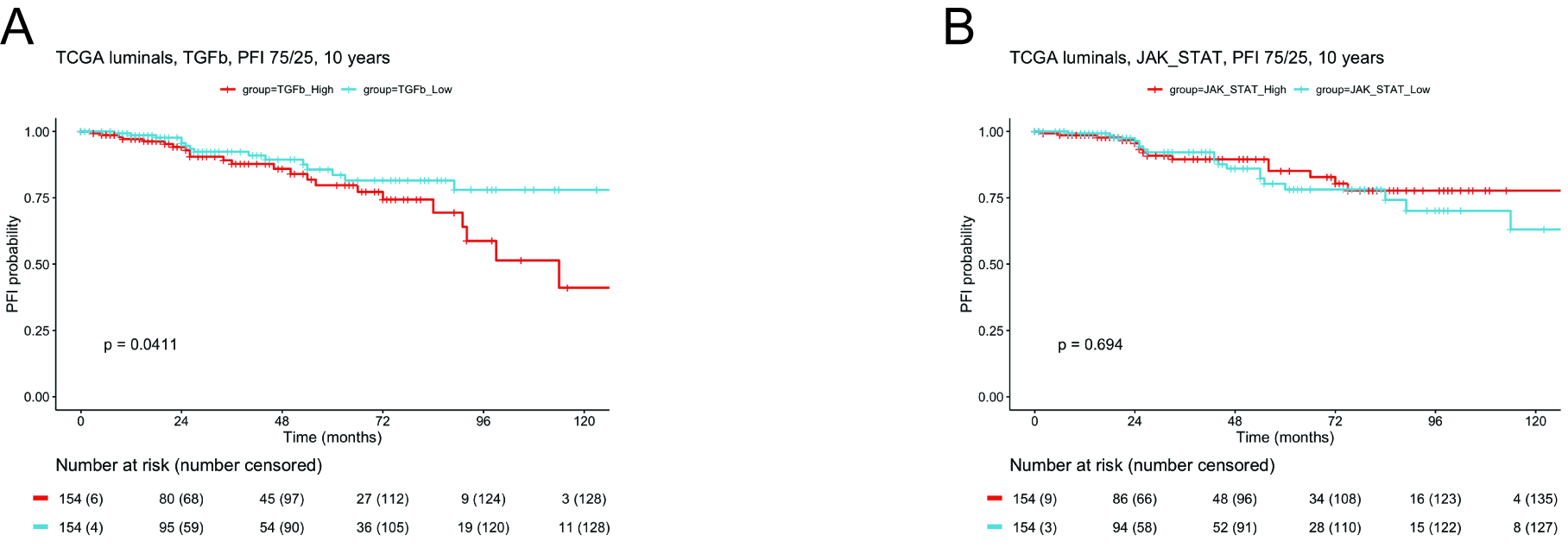

Supplement: Supplementary file 8 — SFig 7 [file 41388_2024_2973_MOESM8_ESM.tif]
