# Supplementary material for: Cancer-associated fibroblasts rewire the estrogen receptor response in luminal breast cancer, enabling estrogen independence
Source: Oncogene. 2024 Feb 22;43(15):1113–26. doi: 10.1038/s41388-024-02973-x (PMC10997519; doi:10.1038/s41388-024-02973-x)
Supplement: Supplementary file 9 — Supplemental figure legends [file 41388_2024_2973_MOESM9_ESM.docx]

**Supplementary figure 1. ER**-α **activity is reduced using two other CAF cell lines.** MCF7 ER-α-reporter cells were directly co-cultured with two isolations of primary human breast CAF cell lines (CAF-A and CAF-B) that were seeded on collagen-coated wells and assessed for luciferase activity (A). Mo = monoculture. Unpaired ordinary one-away ANOVA, Fisher’s LSD multiple comparisons test: ****P<0.0001. Determination of ER-α protein expression by Western blot of cell lysates from MCF7 mono-cultures, co-cultures with CAF2 cells, or CAF2 cells alone (B). Signal intensity was quantified and normalized against the reference GAPDH (C).

**Supplementary figure 2. CAFs do not affect HER2 expression level in BT474 cells.** The estrogen induced reduction of HER2 in BT474 clone 5 cancer cells is maintained in CAF2 cocultures and not affected by secreted factors. CTL = control; CM = conditioned media. Unpaired ordinary one-away ANOVA, Fisher’s LSD multiple comparisons test: *P<0.05, **P<0.01, ***P<0.001. Error bars: SEM.

**Supplementary figure 3. RNA seq of MCF7 cells cocultured with CAFs.** Schematic representation of the RNA-seq experiment at both timepoints of estrogen stimulation (A). Scatter plot showing gene expression regulation by either CAF2, E2 or both at 24h (B). Scatter plot showing gene expression regulation by either CAF2, E2 or both at 6h (C).

**Supplementary figure 4. Drug screen of MCF7 cells cocultured with CAFs.** Schematic representation of the drug screen experiment showing incubation times with CAF2, drugs and estrogen before analysis.

**Supplementary figure 5. The myofibroblast phenotype is dynamically regulated by malignant cells.**  Immunostaining of CAF2 cells for α-smooth muscle actin (red) as a surrogate marker for myofibroblasts. The specificity of the signal is demonstrated by omission of the primary antibody (A). Whereas co-culturing with MCF7 cells induces the myofibroblast phenotype in CAF2 cells (B-C), inhibition of TGF-β or JAK with the inhibitors SB-431542 (D-E) or Pyridone 6 (F-G), respectively, reduces expression of α-smooth muscle actin (H).

**Supplementary figure 6. TGF-β or JAK inhibition causes differential effects in ER-α^+^ breast cancer cell lines.** The effect of TGF-β type I receptor inhibition and JAK inhibition on T47D (A-B) or BT474 (C-D) ERE-activity in mono-cultures or in co-cultures with CAF2 cells was validated through the use of the TGF-β type I receptor (SB-431542; TGF-βi, 5 µM) or the pan-JAK inhibitor Pyridone 6 (JAKi, 75nM).

**Supplementary figure 7. TGF-β pathway activity is associated with poor response to endocrine therapy.** Kaplan-Meier analysis of progression-free interval of luminal breast cancer patients from the TCGA cohort dichotomized for the 25% of patients with the highest TGF-β (A) or JAK (B) pathway activity scores *vs* the 25% of patients with the lowest score.
